# Comparative Performance of Genomic Methods for the Detection of Pyrazinamide Resistance and Heteroresistance in Mycobacterium tuberculosis

**DOI:** 10.1128/JCM.01907-21

**Published:** 2022-01-19

**Authors:** Michael G. Whitfield, David M. Engelthaler, Christopher Allender, Megan Folkerts, Tim H. Heupink, Jason Limberis, Robin M. Warren, Annelies Van Rie, John Z. Metcalfe

**Affiliations:** a National Institute for Health Research, Health Protection Research Unit in Healthcare Associated Infections and Antimicrobial Resistance, Imperial College London, London, UK; b Family Medicine and Population Health (FAMPOP), Faculty of Medicine and Health Sciences, University of Antwerp, Antwerp, Belgium; c South African Medical Research Council Centre for Tuberculosis Research/DSI-NRF Centre of Excellence for Biomedical Tuberculosis Research, Division of Molecular Biology and Human Genetics, Stellenbosch University, South Africa; d Translational Genomics Research Institute, Flagstaff, Arizona, USA; e University of California San Francisco, San Francisco, California, USA; University of Manitoba

**Keywords:** pyrazinamide, resistance, *pncA*, sequencing, *Mycobacterium tuberculosis*

## Abstract

Pyrazinamide is an important component of both drug-susceptible and drug-resistant tuberculosis treatment regimens. Although approximately 50% of rifampin-resistant isolates are also resistant to pyrazinamide, pyrazinamide susceptibility testing is not routinely performed due to the challenging nature of the assay. We investigated the diagnostic accuracy of genotypic and phenotypic methods and explored the occurrence of pyrazinamide heteroresistance. We assessed pyrazinamide susceptibility among 358 individuals enrolled in the South African EXIT-RIF cohort using Sanger and targeted deep sequencing (TDS) of the *pncA* gene, whole-genome sequencing (WGS), and phenotypic drug susceptibility testing. We calculated the diagnostic accuracy of the different methods and investigated the prevalence and clinical impact of *pncA* heteroresistance. True pyrazinamide susceptibility status was assigned to each isolate using the Köser classification and expert rules. We observed 100% agreement across genotypic methods for detection of *pncA* fixed mutations; only TDS confidently identified three isolates (0.8%) with minor variants. For the 355 (99.2%) isolates that could be assigned true pyrazinamide status with confidence, phenotypic DST had a sensitivity of 96.5% (95% confidence interval [CI], 93.8 to 99.3%) and specificity of 100% (95% CI, 100 to 100%), both Sanger sequencing and WGS had a sensitivity of 97.1% (95% CI, 94.6 to 99.6%) and specificity of 97.8% (95% CI, 95.7 to 99.9%), and TDS had sensitivity of 98.8% (95% CI, 97.2 to 100%) and specificity of 97.8% (95% CI, 95.7 to 99.9%). We demonstrate high sensitivity and specificity for pyrazinamide susceptibility testing among all assessed genotypic methods. The prevalence of pyrazinamide heteroresistance in Mycobacterium tuberculosis isolates was lower than that identified for other first-line drugs.

## INTRODUCTION

The World Health Organization (WHO) estimates that 465,000 new cases of rifampin-resistant TB (RR-TB) occurred in 2019, of which the majority had concurrent isoniazid resistance (i.e., multidrug-resistant TB [MDR-TB]) (1). MDR-TB has a high mortality rate and is responsible for about one-third of deaths related to antimicrobial resistance globally (2). Rapid identification and initiation of effective treatment regimens is crucial for improving the treatment outcome of patients with RR-TB.

Pyrazinamide (PZA), a prodrug that is hydrolyzed to its active form, pyrazinoic acid (POA), by the *pncA*-encoded pyrazinamidase, is a strong sterilizing component of the current treatment regimens for both drug-susceptible and drug-resistant TB (1, 3–6). PZA is likely to continue to play a crucial role in TB treatment as evidenced by its inclusion in many regimens assessed in ongoing clinical trials (1; https://clinicaltrials.gov/ct2/show/NCT04311502). While PZA monoresistance occurs in fewer than 3% of isoniazid (INH) and rifampin (RIF)-susceptible strains, high rates of PZA resistance have been noted in MDR-TB (∼50%) (7, 8). PZA-resistant TB may also promote amplification to pre-extensively drug-resistant TB (XDR-TB) when PZA is included in second-line treatment in the presence of undetected PZA resistance (9). Nevertheless, PZA drug susceptibility testing (DST) is rarely performed prior to initiation of PZA-containing regimens (7, 8, 10) due to the complexity of culture-based phenotypic PZA DST.

Since the 1990s, it has been known that mutations in the Mycobacterium tuberculosis-specific *pncA* gene are the primary determinants of PZA resistance, as these *pncA* variants often lead to decreased pyrazinamidase activity (11–15). Sequencing of *pncA* has 87 to 95% sensitivity and 93% to 99% specificity for detection of PZA resistance compared to phenotypic DST methods (7, 8). Imperfect sensitivity may be explained by resistance caused by variants in other genes, such as *panD* (16, 17), *rspA* (18, 19), and *clpC1* (20–22), or by heteroresistance (23). Suboptimal specificity is due to the occurrence of synonymous mutations in *pncA* and errors in the technically challenging phenotypic DST methods (24, 25). Only recently was a commercially assay line probe assay (Nipro Genoscholar PZA-TB [Osaka, Japan]) approved by the WHO to rapidly detect PZA resistance (26–29).

In order to advance the development of rapid genotypic DST for PZA, we assessed the performance of key genotypic methods in identifying resistance-associated variants (RAVs) in the *pncA* gene and promoter region among RR-TB patients in South Africa. Accordingly, the primary aims of the study were to compare the diagnostic accuracy of single-molecule overlapping read (SMOR) targeted deep sequencing (TDS) (30), Sanger sequencing, and whole-genome sequencing (WGS). In addition, we explored the prevalence of PZA heteroresistance in M. tuberculosis isolates and describe the PZA phenotype of PZA-heteroresistant isolates and the impact of the presence of PZA heteroresistance on treatment outcome.

## MATERIALS AND METHODS

### Clinical isolates.

Sputum samples were collected through a prospective cohort (EXIT-RIF study) of patients diagnosed with RR-TB by MTBDR*plus* (Hain Lifescience, Nehren, Germany) or Xpert MTB/RIF (Cepheid Inc., Sunnyvale, CA, USA) between November 2012 and December 2013 in three South African provinces (Free State, Eastern Cape, and Gauteng) (31).

### Laboratory procedures.

Sputum samples were cultured in MGIT media (BD Diagnostic Systems, NJ, USA) following decontamination with *N*-acetyl-l-cysteine–sodium hydroxide (NALC-NaOH). Phenotypic susceptibility to PZA was performed using the nonradiometric MGIT 960 method, which makes use of modified test media to support the growth at a pH of 5.9. A critical concentration of 100 μg/ml PZA was used to distinguish between resistant and susceptible isolates (32). MIC testing was used to determine resistance at PZA concentrations of 25, 50, 70, 100, and 200 μg/ml.

DNA was extracted from cultured isolates using the phenol-chloroform extraction method followed by ethanol precipitation (33).

### Sanger sequencing and bioinformatic analysis.

The entire *pncA* gene (561 bp), as well as 80 bp of the upstream sequence, was Sanger sequenced according to the method of Streicher et al. (34). PCR products were electrophoresed on an ABI 3730xl genetic analyzer (Applied Biosystems, Foster City, CA, USA). Sequences were analyzed on the BioEdit sequence alignment editor with H37Rv as the reference strain.

### Whole-genome sequencing and bioinformatics analysis.

DNA was prepared for WGS following the method of Baym et al. (35) or using Illumina’s DNA prep kit as per the manufacturer’s instructions. Pooled libraries were sequenced on an Illumina HiSeq 2500 system or an Illumina MiniSeq system as per the manufacturer’s protocol. Reads were quality checked using FastQC and aligned to the reference genome H37Rv (GenBank accession no. NC_000962.3) using BWA-MEM (36). Genome coverage was assessed, and a minimum of 40× median coverage was required for inclusion in the analysis. Joint variant calling was conducted by GATK4 using the MarkDuplicates, HaplotypeCaller, GenotypeGVCFs, and Variant Quality Score Recalibration tools (37).

### Targeted deep sequencing and bioinformatics analysis.

M. tuberculosis DNA was amplified and prepared for targeted sequencing and SMOR analysis as previously described (30). After the initial gene-specific PCR, a single 1.0× AMPure XP bead (Beckman Coulter, Brea, CA, USA) cleanup was performed to remove primer-dimer and remnant PCR artifacts. The *pncA*-specific multiplex reaction contains eight primers that span the gene and promoter region (Table 1), resulting in four overlapping amplicons of similar concentrations. The target for sequence coverage was 40,000 raw reads per amplicon. Numerous “no template” negative-control reactions were included to ensure absence of well-to-well sample contamination. DNA from a pan-susceptible M. tuberculosis strain (H37Ra; ATCC 25177) was used as a positive control for all sequencing runs.

**TABLE 1:** M. tuberculosis *pncA*-specific primers with universal tail sequences^*a*^

Primer (direction)	Sequence	Final concn in PCR reaction (μM)
pncAf-55 (forward)	**ACCCAACTGAATGGAGC**CTGCCGCGTCGGTAGGCAAACTGC	400
pncAf102 (forward)	**ACCCAACTGAATGGAGC**CTGGCCGAAGCGGCGGACTACCATC	75
pncAf223 (forward)	**ACCCAACTGAATGGAGC**GTACTCCCGGCGCGGACTTCCATCC	300
pncAf361 (forward)	**ACCCAACTGAATGGAGC**GGCAACGCGGCGTCGATGAGGTC	200
pncAr238 (reverse)	**ACGCACTTGACTTGTCTTC**CAGTCGCCAGGAGGGCCGCGCC	400
pncAr344 (reverse)	**ACGCACTTGACTTGTCTTC**CTTCCTCAGCTGCTCTTGCCGTGCGG	75
pncAr462 (reverse)	**ACGCACTTGACTTGTCTTC**CCATGCGTTACCGAACCGGTGGTCC	300
pnAr+44 (reverse)	**ACGCACTTGACTTGTCTTC**GACAACCGCCGCCACCTCGCG	200

a Primer sequences are shown for the tiled amplification of the entire *pncA* gene, including the promoter region. Previously published universal tail sequences are highlighted in bold (30).

The previously published TB Amplicon Sequencing Analysis Pipeline (ASAP) (38) was used in conjunction with SMOR analysis to automate the process of sequence alignment and quantify alleles of interest for each overlapping read pair, as previously described (30). The ASAP json file was updated to screen for any variant in the *pncA* gene against the standard H37Rv reference genome (38). Because the SMOR analysis module within ASAP excludes paired reads from the same molecule whose sequences disagree, this method results in a higher level of confidence to call low-level (<1%) variant subpopulations than single-read analysis. In the analysis, the minor variant calling threshold was set to 1% to ensure a very high confidence in heteroresistance calls.

### Data analysis.

We first investigated and resolved any discrepancies among genotypic methods and between genotypic and phenotypic PZA methods. Genotypic-phenotypic discrepant results were defined as isolates that were phenotypically susceptible in the presence of *pncA* gene variants or isolates that were phenotypically resistant but maintained a wild-type *pncA* gene sequence. In the latter case, we conducted PZA MIC to confirm the PZA-resistant phenotype and used WGS to investigate the occurrence of variants in the *panD*, *rpsA*, *clpC1*, *Rv1258c*, *PPE35*, and *Rv3236c* genes, which have been listed by WHO as PZA resistance candidate genes (39). When a *pncA* variant-containing isolate was phenotypically PZA susceptible, we conducted PZA MIC testing to confirm the susceptible phenotype. In addition, we consulted the Köser classification of the variant (40), which incorporates phenotypic DST, MIC, genotypic DST, homoplasy, and interpretative approaches to assign *pncA* variants to one of five levels of confidence for association with resistance as follows: group A, associated with resistance; B, likely associated with resistance; C, inconclusive evidence; D, likely neutral; and E, neutral. We classified isolates containing group A or B *pncA* variants as PZA resistant and isolates containing group D or E *pncA* variants as PZA susceptible, and we excluded those classified as group C from the analysis (40). For *pncA* variants not included in the Köser classification, we applied the expert rules used in the Köser study (40). A true PZA status (resistant/susceptible) was then assigned to each isolate and used as the reference to determine the diagnostic accuracy (sensitivity, specificity, positive predictive value [PPV], and negative predictive value [NPV]) of the SMOR TDS, Sanger sequencing, WGS, and phenotypic DST.

To determine the prevalence of minor *pncA* variants, variant calling thresholds were set at 1% for TDS (30) and 10% for WGS, as WGS cannot confidently distinguish minor variants from sequencing error at lower thresholds (41, 42). The association between genomic PZA heteroresistance and phenotypic PZA resistance was assessed. TB treatment outcome data were reviewed for patients whose isolate contained minor (<10%) *pncA* variants to investigate the clinical relevance of PZA heteroresistance.

All statistical analyses were performed with R Studio (https://www.R-project.org; Foundation for Statistical Computing, Vienna, Austria).

### Power estimation.

Based on an expected 50% prevalence of PZA resistance in RR-TB patients and an expected point estimate for sensitivity of 90% for detection of PZA resistance, the sample of 358 culture isolates would achieve a small (4%) margin of error for the sensitivity of PZA detection by genotypic methods (43).

### Ethics.

All patients gave written informed consent for participation in the EXIT-RIF cohort study and DST and genotypic analysis of their culture isolates. The study was approved by the institutional review board of the University of North Carolina, Chapel Hill, United States, the Human Ethics Research Committee of the University of the Witwatersrand, Johannesburg, South Africa, and the Stellenbosch University Health Research Ethics Committee (reference no. S12/01.020).

## RESULTS

### Cohort description.

Among the 749 participants from the EXIT-RIF study, 389 (52%) had a history of TB treatment, and 581 (77.6%) were HIV positive. M. tuberculosis culture isolates were available for phenotypic PZA DST, WGS, Sanger sequencing of the *pncA* gene and its promoter region, and TDS of the *pncA* gene for 358 (47.8%) participants, of which 243 (68%) were MDR-TB. Phenotypic MGIT 960 DST classified 169 (47.2%) of the 358 isolates as resistant to PZA at a critical concentration of 100 μg/ml.

### Genotypic results.

**FIG 1:**
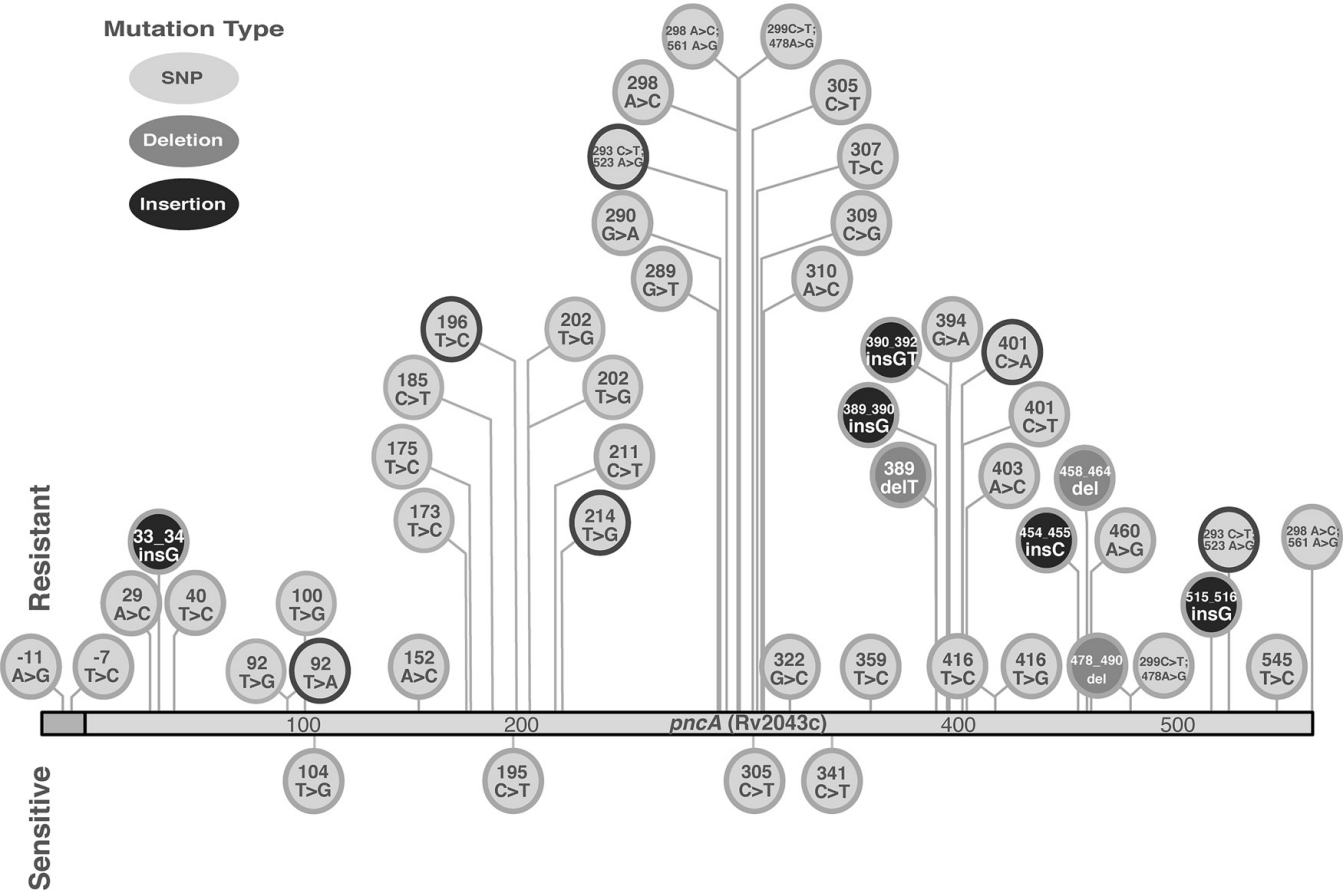
Distribution of *pncA* variants across the entire *pncA* gene, with resistant-associated variants shown on top and susceptible-associated variants on the bottom.

Sanger sequencing and WGS both classified 178 (49.7%) isolates as wild type for the *pncA* gene and detected the following same variants in 180 (50.3%) isolates: a single nucleotide polymorphism (SNP) (*n* = 89), a frameshift variant (insertion or deletion [indel]) (*n* = 80), a double *pncA* variant (*n* = 9), and a *pncA* promoter variant (*n* = 2). In total, 48 distinct *pncA* variants (40 SNPs and 8 indels) were observed (Fig. 1). By WGS, all identified variants were fixed (>90% of the M. tuberculosis population) without evidence of heteroresistance.

TDS (average 34,392 reads/amplicon) classified 175 (48.9%) of the isolates as wild type for the *pncA* gene while identifying the same fixed variants reported by WGS and Sanger sequencing in 180 isolates. In addition, three isolates (0.8%; 95% confidence interval [CI], 0.3 to 2.4%) contained a minor *pncA* variant. These isolates included a Q10P mutation, an A134V mutation, and a W68G mutation present at 2%, 3%, and 7%, respectively, of the total M. tuberculosis population. These minor variants were not detected by Sanger sequencing or WGS.

### Investigation of discrepancies between tests and assignment of PZA status.

Genotype-phenotype discrepancies were observed in 14 of the 358 isolates (3.9%). Four isolates were phenotypically resistant without demonstrated *pncA* RAVs, and seven isolates were phenotypically susceptible but contained a fixed *pncA* variant (Table 2). The three heteroresistant isolates were phenotypically susceptible.

**TABLE 2:** Summary of results of different methods to detect variants in the *pncA* gene

No. of samples	*pncA* Sanger result	*pncA* WGS result	TDS result (% of reads)	PZA DST result	True PZA status	Comment
171	Wild type	Wild type	Wild type	Susceptible	Susceptible	
1	Wild type	Wild type	29 A→C (2.03)	Susceptible	Resistant	Minor variant
1	Wild type	Wild type	202 T→G (6.7)	Susceptible	Resistant	Minor variant
1	Wild type	Wild type	401 C→T (3.06)	Susceptible	Resistant	Minor variant
2	Wild type	Wild type	Wild type	Resistant (>100, ≤200)	Resistant	Discordant genotype/phenotype
2	Wild type	Wild type	Wild type	Resistant	Resistant	Discordant genotype/phenotype
1	−11 A→G	−11 A→G	−11 A→G (99.98)	Resistant	Resistant	
1	−7 T→C	−7 T→C	−7 T→C (99.95)	Resistant	Resistant	
1	29 A→C	29 A→C	29 A→C (99.97)	Resistant	Resistant	
5	Frameshift 33C→CG	Frameshift 33C→CG	Frameshift 33 C→CG (≥98.4)	Resistant	Resistant	
10	40 T→C	40 T→C	40 T→C (≥99.93)	Resistant	Resistant	
1	92 T→A	92 T→A	92 T→A (99.88)	Resistant	Resistant	
2	92 T→G	92 T→G	92 T→G (≥99.92)	Resistant	Resistant	
1	100 T→G	100 T→G	100 T→G (99.96)	Resistant	Resistant	
3	104 T→G	104 T→G	104 T→G (≥98.89)	Susceptible (>25, <75)	Susceptible	Neutral mutation (40)
2	152 A→C	152 A→C	152 A→C (≥99.7)	Resistant	Resistant	
1	173 T→C	173 T→C	173 T→C (99.96)	Resistant	Resistant	
4	175 T→C	175 T→C	175 T→C (≥99.85)	Resistant	Resistant	
1	185 C→T	185 C→T	185 C→T (99.98)	Resistant	Resistant	
8	195 C→T	195 C→T	195 C→T (≥98.16)	Susceptible	Susceptible	Silent mutation
1	196 T→C	196 T→C	196 T→C (99.94)	Resistant	Excluded	
1	202 T→C	202 T→C	202 T→C (99.95)	Resistant	Resistant	
2	202 T→G	202 T→G	202 T→G (≥99.84)	Resistant	Resistant	
4	211 C→T	211 C→T	211 C→T (≥99.94)	Resistant	Resistant	
1	214 T→G	214 T→G	214 T→G (99.96)	Susceptible (≤75)	Resistant	Not classified (40)
3	289 G→T	289 G→T	289 G→T (≥99.97)	Resistant	Resistant	
9	290 G→A	290 G→A	290 G→A (≥99.94)	Resistant	Resistant	
1	293 C→T + 523 A→G	293 C→T + 523 A→G	293 C→T (99.95) + 523 A→G (99.97)	Resistant	Resistant	
1	298 A→C	298 A→C	298 A→C (99.96)	Resistant	Resistant	
4	298 A→C + 561 A→G	298 A→C + 561 A→G	298 A→C (≥99.79) + 561 A→G (≥99.84)	Resistant	Resistant	
4	299 C→T + 478 A→G	299 C→T + 478 A→G	299 C→T (≥99.94) + 478 A→G (≥99.62)	Resistant	Resistant	
1	305 C→T	305 C→T	305 C→T (99.97)	Resistant	Resistant	
2	305 C→T	305 C→T	305 C→T (≥99.94)	Susceptible (>25 < 75)	Resistant	Resistant (40)
2	307 T→C	307 T→C	307 T→C (≥99.93)	Resistant	Resistant	
2	309 C→G	309 C→G	309 C→G (≥99.78)	Resistant	Resistant	
1	310 A→C	310 A→C	310 A→C (99.94)	Resistant	Resistant	
1	322 G→C	322 G→C	322 G→C (99.9)	Resistant	Resistant	
1	341 C→T	341 C→T	341 C→T (99.98)	Susceptible (<25)	Susceptible	Likely neutral mutation (40)
1	359 T→C	359 T→C	359 T→C (99.9)	Resistant	Resistant	
2	Frameshift 389 T→_	Frameshift 389 T→_	Frameshift 389 T→_ (≥94.87)	Resistant	Resistant	
1	Frameshift 389 T→TG	Frameshift 389 T→TG	Frameshift 389 T→TG (93.26)	Resistant	Resistant	
1	Frameshift 390 G→GGT	Frameshift 390 G→GGT	Frameshift 390 G→GGT (95.75)	Resistant	Resistant	
1	394 G→A	394 G→A	394 G→A (99.82)	Resistant	Resistant	
1	395 G→C	395 G→C	395 G→C (99.94)	Resistant	Resistant	
1	401 C→A	401 C→A	401 C→A (≥99.96)	Resistant	Resistant	
2	401 C→T	401 C→T	401 C→T (≥99.91)	Resistant	Resistant	
6	403 A→C	403 A→C	403 A→C (≥99.71)	Resistant	Resistant	
1	416 T→C	416 T→C	416 T→C (99.92)	Resistant	Resistant	
3	416 T→G	416 T→G	416 T→G (≥99.94)	Resistant	Resistant	
3	Frameshift 454 G→GC	Frameshift 454 G→GC	Frameshift 454 G→GC (95.13)	Resistant	Resistant	
1	Frameshift 458 (8 bp del)	Frameshift 458 (8 bp del)	Frameshift 458 (8 bp del) (≥99.93)	Resistant	Resistant	
6	460 A→G	460 A→G	460 A→G (99.92)	Resistant	Resistant	
3	Frameshift 478 A→ACAGCGGGTGTG	Frameshift 478 A→ACAGCGGGTGTG	Frameshift 478 A→ACAGCGGGTGTG (≥97.47)	Resistant	Resistant
64	Frameshift 515 T→TG	Frameshift 515 T→TG	Frameshift 515 T→TG (≥92.15)	Resistant	Resistant	
1	545 T→C	545 T→C	545 T→C (99.96)	Resistant	Resistant	

For the four phenotypically resistant isolates without demonstrated *pncA* RAV, all were wild type for *panD*, *rpsA*, *Rv1258c*, *PPE35*, and *Rv3236c*; one isolate demonstrated a previously undescribed *clpC1* synonymous variant (N806N). MIC results confirmed resistance phenotype in two isolates (MIC 100 to 200 μg/ml); the other two could not be tested due to contamination. A PZA-resistant status was assigned to the two confirmed resistant isolates, and the two contaminated isolates were excluded from the accuracy analysis.

For the seven phenotypically susceptible isolates with fixed *pncA* variants, our MIC results confirmed the susceptible phenotype in all seven isolates (MIC below the critical concentration of 100 μg/ml). Four different SNPs in the *pncA* gene occurred in the seven susceptible isolates L35R (*n* = 3), C72G (*n* = 1), A102V (*n* = 2), and T114M (*n* = 1). The L35R and T114M variants are considered PZA susceptible per the Köser classification (groups E and D, respectively) (40). Consequently, a PZA-susceptible status was assigned to the four isolates containing the L35R or T114M variant. Because the A102V variant is classified as PZA resistant by the Köser classification (group B) (40), the two isolates containing an A102V variant were assigned a PZA-resistant status for the accuracy analysis. The C72G variant is not included in the Köser classification list but is deemed PZA resistant according to the expert rule (40).

The three isolates containing minor variants (Q10P, A134V, and W68G) were all phenotypically susceptible, consistent with the phenotypic PZA DST limit of detection of 10% (32). Because these variants are associated with PZA resistance by Köser classification (class A and B) (40), we classified these isolates as resistant (40). The treatment outcomes of three patients with heteroresistant M. tuberculosis isolates were as follows: the patient with RIF-monoresistant M. tuberculosis with an A134V mutation defaulted treatment, and the two patients with isolates demonstrating Q10P and W68G mutations successfully completed PZA-containing second-line treatment (as defined by culture conversion). The patient with MDR-TB with a Q10P mutation (2%) received PZA for 24 months; the patient with drug-susceptible M. tuberculosis demonstrating a W68G mutation (7%) received PZA for 13 months. Unfortunately, there were no serial isolates obtained for these patients.

Overall, we could assign a true PZA status to 355 of 358 (99.2%) isolates as follows: 183 of the 358 isolates were classified PZA susceptible (171 isolates wild type, 8 isolates with a silent mutation, and 4 isolates with a *pncA* variant that does not confer resistance). One hundred seventy-two of 358 (48.0%) isolates were classified as PZA resistant (170 isolates with demonstrated RAVs [including the three isolates with resistance-associated minor variants detected by TDS] and 2 isolates phenotypically resistant but genotypically wild type). Three isolates were excluded: one isolate could not be classified with confidence due to the presence of a variant of unknown phenotype (Köser group C) (40), and two isolates were phenotypically PZA resistant without a *pncA* variant but unable to undergo MIC confirmation due to contamination.

### Diagnostic accuracy of DST methods.

Based on the results of the 355 isolates for which we could assign a PZA status with high confidence, phenotypic DST had a sensitivity of 96.5% (95% CI, 93.8 to 99.3) and specificity of 100% (95% CI, 100 to 100), with a PPV of 100% and NPV of 96.8%. Sanger sequencing and WGS both has a sensitivity of 97.1% (95% CI, 94.6 to 99.6) and specificity of 97.8% (95% CI, 95.7 to 99.9), a PPV of 97.7%, and an NPV of 97.3%. SMOR TDS had a sensitivity of 98.8% (95% CI, 97.2 to 100) and specificity of 97.8% (95% CI, 95.7 to 99.9), a PPV of 97.7%, and an NPV of 98.9%. A sensitivity analysis, including the two isolates which were *pncA* wild type but phenotypically PZA resistant assigned as PZA resistant, or the three isolates with resistant minor variants but with susceptible phenotype reassigned as PZA susceptible, did not significantly change the diagnostic accuracy of assessed phenotypic or genotypic methods.

## DISCUSSION

Using a multifaceted reference standard, we demonstrate a high diagnostic accuracy for the detection of PZA resistance for SMOR TDS (sensitivity, 98.8% [95% CI, 97.2 to 100]); specificity, 97.8% [95% CI, 95.7 to 99.9%]), as well as conventional genotypic methods (Sanger sequencing and WGS, sensitivity 97.1% [95% CI, 94.6 to 99.6%]; specificity, 97.8% [95% CI, 95.7 to 99.9%]), and phenotypic DST (sensitivity, 96.5% [95% CI, 93.8 to 99.3%]; specificity, 100% [95% CI, 100 to 100%]). We believe these estimates are highly accurate, as we confirmed standard phenotypic DST with MIC testing in an experienced laboratory and reclassifying silent and neutral *pncA* mutations as susceptible using the Köser classification (40). SMOR TDS showed a slightly higher sensitivity than Sanger and WGS due to the ability of the technique to identify the presence of minor variants below 10% of the total mycobacterial population.

The diagnostic accuracy of the genomic DST methods assessed was higher than that reported for Sanger sequencing in two studies from China, which reported a sensitivity of 78% and 90% (44, 45). The sensitivity and specificity were also higher than that of the high-resolution melt (HRM) methods (89% sensitivity and 82% specificity) (46); the heteroduplex HRM method had a slightly lower sensitivity (97%) and similar specificity (96%) compared to what we observed in our study (46). The linear after the exponential (LATE) PCR method, which uses fluorescent probes to detect the presence of mutations in the *pncA* gene, also reported a slightly lower sensitivity (97%) and similar specificity (97%) (47). A recent WHO-commissioned systematic review was conducted to investigate the diagnostic accuracy of the commercial Nipro PZA line probe assay (LPA) in comparison to other methods to detect PZA resistance (29). A pooled sensitivity of 81.2% and specificity of 97.8% using PZA DST as a reference and a pooled sensitivity of 96.4% and specificity of 100% using genotypic detection as a reference were reported (29). While these are promising results, the LPA technique does not allow the detection of exact *pncA* mutation or insight into possible heteroresistance.

Fourteen isolates (3.9%) had discrepant genotype-phenotype results for PZA susceptibility. Several studies have described a small minority of isolates as phenotypically resistant without an observed *pncA* variant (44, 45, 48–50). Previous investigators have attributed this false-positive resistance to the technical challenges associated with phenotypic DST or to another mechanism of resistance (e.g., resistance-associated variants in *panD*, *rpsA*, and *clpC1*). In two of four isolates, we clearly observe a high MIC (>100 μg/ml) coupled with excellent sensitivity and specificity; in these cases, a false-positive phenotypic DST is highly unlikely. Therefore, an alternative mechanism is likely operative, though we failed to observe any explanative variants in genes previously associated with PZA resistance (39). A genome-wide association study (GWAS) approach to discover novel resistance-associated genes in larger data sets may further enhance our understanding of alternative mechanisms of PZA resistance.

In addition, we observed four *pncA* variants (L35R, C72G, A102V, and T114M) associated with phenotypically susceptible isolates (<100 μg/ml). While L35R and T114M have been previously associated with susceptibility (40), A102V is associated with resistance in the Köser classification (40). Interestingly, examination of the supplemental data reveals a mix of resistant and susceptible liquid cultures, which may suggest that this variant’s PZA MIC may be close to the threshold of 100 μg/ml. Finally, although C72G is not included in the Köser classification, we observed a MIC of ≤75 μg/ml; this may therefore be a false-susceptible phenotypic DST result or a variant with a critical concentration close to the threshold.

The SMOR TDS method identified minor variants that remained undetected by Sanger sequencing, WGS, and even phenotypic DST. While the clinical relevance of PZA heteroresistance less than 10% of the total M. tuberculosis population could not be determined in our study due to the low number (*n* = 3) of cases and lack of serial isolates, studies examining other anti-TB drug classes have suggested that detecting these subpopulations before and during treatment may be clinically relevant (23, 51, 52). The prevalence of PZA minor variants was less than that observed for other anti-TB drugs (e.g., fluoroquinolone microheteroresistance [<5%] occurs among 3/66 of unselected isolates) (23). We hypothesize that among RR-TB patients, this lower prevalence was due to primary transmission of already PZA-resistant strains; conventional selective determinants of microheteroresistant subpopulations (i.e., fitness cost, drug-specific mutation rates [53] and drug exposure and mechanism) are likely similarly important in the earlier phases of PZA resistance acquisition.

Compared to the LPA and LATE PCR assays, the SMOR TDS approach has added value, as it identifies all variants in the *pncA* gene. This is critically important since not all mutations in *pncA* are associated with resistance (40, 54). The targeted approach also presents some limitations vis-à-vis WGS by focusing on preidentified targets (i.e., *pncA* variants), given there are several other candidate genes for PZA resistance (e.g., *panD* [16, 17], *rspA* [18, 19], *clpC1* [20–22], *Rv1258c*, *PPE35*, and *Rv3236c* [39]); however, the majority of these emanate from *in vitro* studies with little support from clinical isolates. In our study, only one of the four phenotypically resistant isolates with the wild-type *pncA* gene had a variant in one of these genes, but this variant (in *clpC1*) was a synonymous SNP and therefore most likely does not explain the phenotype of the isolate. It is possible that there are other unknown mechanisms of resistance that occur at extremely low frequencies. Additional validated non-*pncA* mutations associated with PZA resistance can be included in future TDS assays.

Several limitations to the study should be noted. First, we could not confidently identify the “true” PZA resistance profile in two discordant isolates because the phenotypic status could not be confirmed by MIC. While exclusion of these isolates in the analysis may have overestimated concordance between the genomic assays, a sensitivity analysis revealed that inclusion of these isolates as discordant did not change the results. Second, the prevalence of heteroresistance could have been underestimated, as TDS was done on culture isolates, which may have introduced a culture bias (52). Third, the amplification step of TDS may cause an uneven amplification of subpopulations, which may further introduce bias in the observed heteroresistance. We believe that this may have been minimal as primer concentrations were modified to ensure equal concentrations of all amplicons.

**Conclusions.** Given the central place for PZA in both drug-susceptible and drug-resistant TB regimens, development of an accurate molecular assay for PZA resistance is a high priority. This study demonstrates the high accuracy of the genomic methods to identify variants in the *pncA* gene and determine PZA resistance; in addition, targeted deep sequencing of *pncA* identified the presence of minor variants, the clinical importance of which remains to be validated. While WGS could, in theory, explore the role of other genes in PZA resistance, we were not able to confirm a role of any of the other candidate genes (*panD*, *rpsA*, *clpC1*, *Rv1258c*, *PPE35*, and *Rv3236c*) in clinical PZA resistance. Future large multicountry studies should be performed using culture-free genotypic DST to accurately estimate the prevalence and clinical relevance of *pncA* heteroresistance and the clinical role of genes other than *pncA* in PZA resistance.
